# Visible light leaves evaporation and interfacial structure of neat water unchanged at the air–water interface

**DOI:** 10.1073/pnas.2615377123

**Published:** 2026-09-14

**Authors:** Yucong Chen, Joseph C. Shirley, Zi Xuan Ng, Yongkang Wang, Yuki Nagata, Arsh S. Hazrah, Mischa Bonn

**Affiliations:** ^a^ Department of Molecular Spectroscopy, Max Planck Institute for Polymer Research, Mainz 55128, Germany

**Keywords:** evaporation, photomolecular effect, solar absorption, water structure, interface

## Abstract

Visible light-induced “photomolecular” enhancement of water evaporation has been proposed as a fundamentally new mode of light–matter interaction at aqueous interfaces, with far-reaching implications for climate processes and light-driven water technologies. Yet, the response of neat interfacial water to visible photons has not been directly quantified at both macroscopic and molecular scales. Here, we combine high-precision confocal displacement measurements with a surface-specific vibrational probe that detects the OH-stretch vibrations of interfacial water molecules with subnanometer depth sensitivity, allowing us to resolve even subtle blue shifts of the hydrogen-bonded continuum and changes in the amplitude and position of the free-OH peak that report on interfacial hydrogen-bond strength and molecular orientation. This approach enables us to track both evaporation kinetics and molecular structure at the air–water boundary. Continuous 450, 532, and 635 nm illumination leaves the macroscopic evaporation rate unchanged over a wide range of humidities, while the interfacial vibrational spectra show no detectable modification of water hydrogen-bonding or molecular orientation under identical environmental conditions. To directly test a nonthermal, field-driven mechanism, we further exposed the interface to femtosecond visible and near-infrared pulses spanning 515, 800, and 1030 nm; the peak intensities were increased by more than ten orders of magnitude, and no structural change was observed. Together, these measurements show that the neat air–water interface is remarkably insensitive to visible irradiation, implying that any substantial light-enhanced evaporation is most consistent with photothermal or geometric effects in complex materials rather than from a new, nonthermal and nonresonant pathway intrinsic to liquid water.

## Significance

Visible light has been proposed to drive a new form of “photomolecular” evaporation at water surfaces, but the actual sensitivity of neat interfacial water to visible photons remains unknown. Here, we directly quantify how visible illumination influences both the macroscopic evaporation rate and the molecular structure of the pure water surface. Using precise height tracking of the water surface and an advanced probe of the structure of interfacial water molecules with Ångström-scale depth sensitivity, we find that visible light does not measurably alter either evaporation kinetics or interfacial hydrogen bonding, even at extreme photon fluxes. The air–water interface is robust to visible irradiation, setting strict physical limits on any nonthermal, light-driven evaporation pathway intrinsic to liquid water.

Evaporation plays a central role in both natural and engineered systems. At the planetary level, it governs the hydrological cycle (1), mediates heat exchange between Earth’s surface and the atmosphere (2), and shapes weather and climate patterns (3). At the technological level, evaporation underpins a rapidly expanding class of water-harvesting (4, 5) and energy-conversion platforms (6), including solar vapor generation, where maximizing phase-change efficiency is essential for sustainable desalination and purification (7, 8). As evaporation is tightly coupled to the energetics and molecular structure of interfacial water (9–11), a physically grounded understanding of how external perturbations interact with this interface is crucial. Any proposed revision to the fundamental mechanism of water evaporation, therefore, carries far-reaching implications for environmental science, energy technologies, and our broader view of phase transitions.

**Fig. 1.**
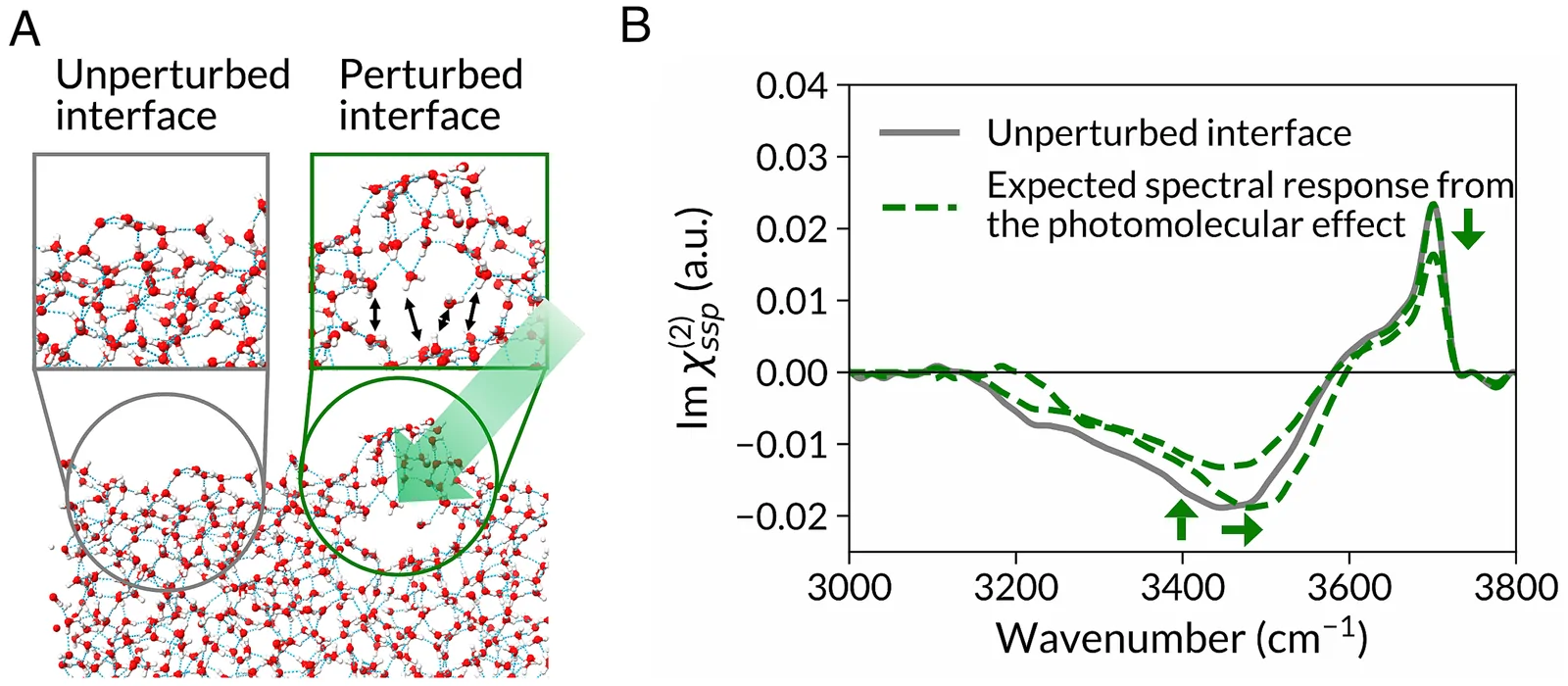
(*A*) Schematic shows the proposed photomolecular effect by Lv et al. (13) and its impact on the hydrogen bond network of interfacial water. Black arrows indicate the breaking of hydrogen bonds due to the proposed photomolecular effect. (*B*) SFG response comparing the unperturbed air/water interface (gray solid line) with the hypothetical response from the interface perturbed by the photomolecular effect (green dashed lines). The green dashed traces highlight the expected amplitude reduction and frequency shifts induced by the photomolecular effect, if present. These expected amplitude changes and spectral shifts are schematic, guided by established analogies, not a quantitative prediction of any specific model (14, 15). See main text for more details.

Recent reports have suggested that visible light can nonthermally enhance water evaporation via a “photomolecular” effect. In these studies, Tu et al. observed evaporation rates that exceed the thermal evaporation limit at fixed visible flux in weakly absorbing hydrogels, even without additional absorbers, with a strong wavelength dependence peaking near 520 nm, and interpreted these results as evidence that visible photons eject nanoscopic water clusters from the air–water interface—the photomolecular effect (12). Lv et al. further proposed that visible photons directly perturb the interfacial hydrogen-bond network, driving cluster emission through a nonthermal, nonresonant pathway, as illustrated in Fig. 1*A* (13). Here, we ask whether neat interfacial water shows any such nonthermal visible-light sensitivity by directly quantifying both the evaporation rate of a flat air–water interface and the accompanying changes—or lack thereof—in the interfacial OH-stretch spectrum under well-defined visible illumination.

Indirect evidence for such a mechanism has been inferred from three types of observations: i) superthermal evaporation rates in illuminated hydrogels with a pronounced wavelength dependence around 520 nm, interpreted as cluster ejection from the air–water interface (12): ii) small differences between “light on” and “light off” IR and Raman spectra recorded above illuminated water, assigned to transient water clusters (13); and iii) nanometer deformations at the apex of illuminated water droplets, attributed to a surface photon process (16). Spectroscopic reassessments have argued that the reported narrow spectral features can be explained by gas-phase water and bulk liquid contributions, and that the signatures do not uniquely require distinct clusters (17). Others have countered that subtle differences persist under specific above-surface geometries and illumination conditions, which they interpret as consistent with light-induced cluster formation near the interface (18). As a result, the existence and origin of a nonthermal, visible-driven evaporation mechanism remain actively debated.

Any proposed nonthermal mechanism by which visible light perturbs evaporation at the neat air–water interface must satisfy stringent physical constraints. Liquid water is essentially transparent across the visible spectrum and lacks low-lying electronic or vibrational states that can resonantly absorb visible photons (19, 20). As a result, any coupling between visible light and interfacial water must proceed through nonresonant field interactions. At the same time, ejecting molecular clusters from the interface would require the cooperative weakening or breaking of multiple hydrogen bonds, with energetic costs far exceeding typical thermal fluctuations at ambient conditions (21–23). Together, these considerations place strong constraints on the magnitude and detectability of any nonthermal visible light–driven interfacial response, and motivate direct, surface-specific tests of interfacial structure and evaporation kinetics.

Existing experimental support for the photomolecular effect is based almost entirely on indirect observables, such as mass loss from porous matrices, open-path IR and Raman spectra that are susceptible to contributions from gas-phase water and bulk liquid that can complicate interpretation, and interferometric droplet deformations that require model-dependent interpretation (13). Importantly, these approaches do not directly probe the hydrogen-bond network of neat interfacial water, making it difficult to disentangle true surface responses from effects originating in the bulk, the surrounding vapor, or the solid matrix. This lack of direct, surface-specific information is a central obstacle to evaluating whether visible photons genuinely perturb the air–water interface. Our central question is whether neat interfacial water exhibits any detectable nonthermal response to visible photons sufficient to contribute to evaporation beyond thermal expectations.

Here, we provide a direct, molecular-level characterization of how visible illumination influences both the kinetics and structure of the neat air–water interface. High-precision confocal displacement measurements quantify the evaporation rate of pure water under well-controlled illumination and humidity conditions. In parallel, we employ surface-specific vibrational spectroscopy (24, 25) that reads out the OH stretch vibrations of interfacial water molecules with approximately Ångström-scale depth sensitivity, allowing us to monitor changes in hydrogen bonding (26, 27), orientation (28, 29), and local electric fields at the interface (30, 31). The theoretical model underlying these reports attributes the excess flux to visible photons cooperatively cleaving hydrogen-bonded water clusters from the interface (32). Such a process is, by construction, an interfacial hydrogen-bond rearrangement: it must deplete the bonded-OH continuum and alter the free-OH population that heterodyne-detected SFG resolves directly. As interfacial hydrogen-bond ordering measured by SFG has been shown to track the evaporation rate itself, a surface-specific spectral measurement constitutes a direct test of the model’s central structural prediction rather than a correlate of it (10). The expected spectral signatures in Fig. 1*B* are guided by established responses of the air–water interface to perturbations that modify hydrogen-bond connectivity. Upon increasing temperature, the average hydrogen-bond coordination number and lifetime at the interface decrease, shifting the population toward less-coordinated, more weakly bound water molecules. This produces a blue shift and amplitude reduction of the hydrogen-bonded OH continuum, with comparatively modest changes in the free-OH feature (14). Complementary insight from vibrational spectroscopy of gas-phase water clusters shows that reducing cluster size and coordination yields analogous spectral shifts, directly linking these responses to changes in local hydrogen-bond topology (33). Similarly, increased interfacial curvature and certain electrolyte solutions reduce the intensity of both the hydrogen-bonded band and the free-OH peak, reflecting a loss of extended hydrogen-bond connectivity and altered orientational order (15). These analogies suggest that any mechanism that ejects nanoscopic clusters or substantially weakens interfacial hydrogen bonding should leave a comparable spectral fingerprint in the SFG response. We note that very low surface coverages or short-lived cluster precursors could, in principle, yield smaller spectral signatures than those schematically illustrated in Fig. 1*B*; however, any mechanism capable of producing evaporation enhancements on the order of 10 to 40% would almost certainly require interfacial hydrogen-bond and free-OH perturbations large enough to be detectable within our SFG sensitivity. Once clusters have grown into nearly symmetric structures and moved away from the interface, their net second-order response can vanish; our SFG measurements therefore primarily probe the interfacial reorganization and transient asymmetries required to create such clusters, rather than any subsequent bulk-like cluster population.

Although the photomolecular effect has been reported under continuous visible illumination, its proposed origin is a nonthermal, field-driven perturbation of the interfacial hydrogen-bond network rather than simple heating. For such a nonresonant mechanism, the response should scale predominantly with instantaneous electric-field strength (and thus photon flux), whereas purely photothermal effects depend on the average absorbed power. Ultrafast visible and near-infrared pulses with peak power densities exceeding 10^10^ W cm^–2^ therefore provide a stringent test: if visible photons directly drive cluster ejection or substantial bond weakening, associated spectral signatures should become more prominent under these conditions. Together, these experiments establish quantitative limits on how visible light can influence both evaporation and molecular structure at the neat air–water interface. These limits, in turn, allow us to critically evaluate proposed photomolecular mechanisms without relying on indirect observables.

## Results and Discussion

### Macroscopic Evaporation Kinetics Remain Unchanged under Visible Irradiation.

If visible light weakens the interfacial hydrogen-bond network and ejects clusters, the net mass flux across a flat interface should increase; we therefore directly track interface recession rather than inferred thermal balances in complex media. To this end, we used a high-precision confocal displacement sensor to continuously track the position of the air–water interface in real time, with and without irradiation. As the water surface recedes due to evaporation, the measured height decrease can be directly converted into an evaporation rate through purely geometric measurements, without relying on indirect thermal assumptions. This measurement geometry directly probes evaporation at a flat, well-defined liquid interface, in contrast to earlier approaches that relied on thermal balances in complex, porous, or optically scattering systems, where evaporation rates must be inferred indirectly.

**Fig. 2.**
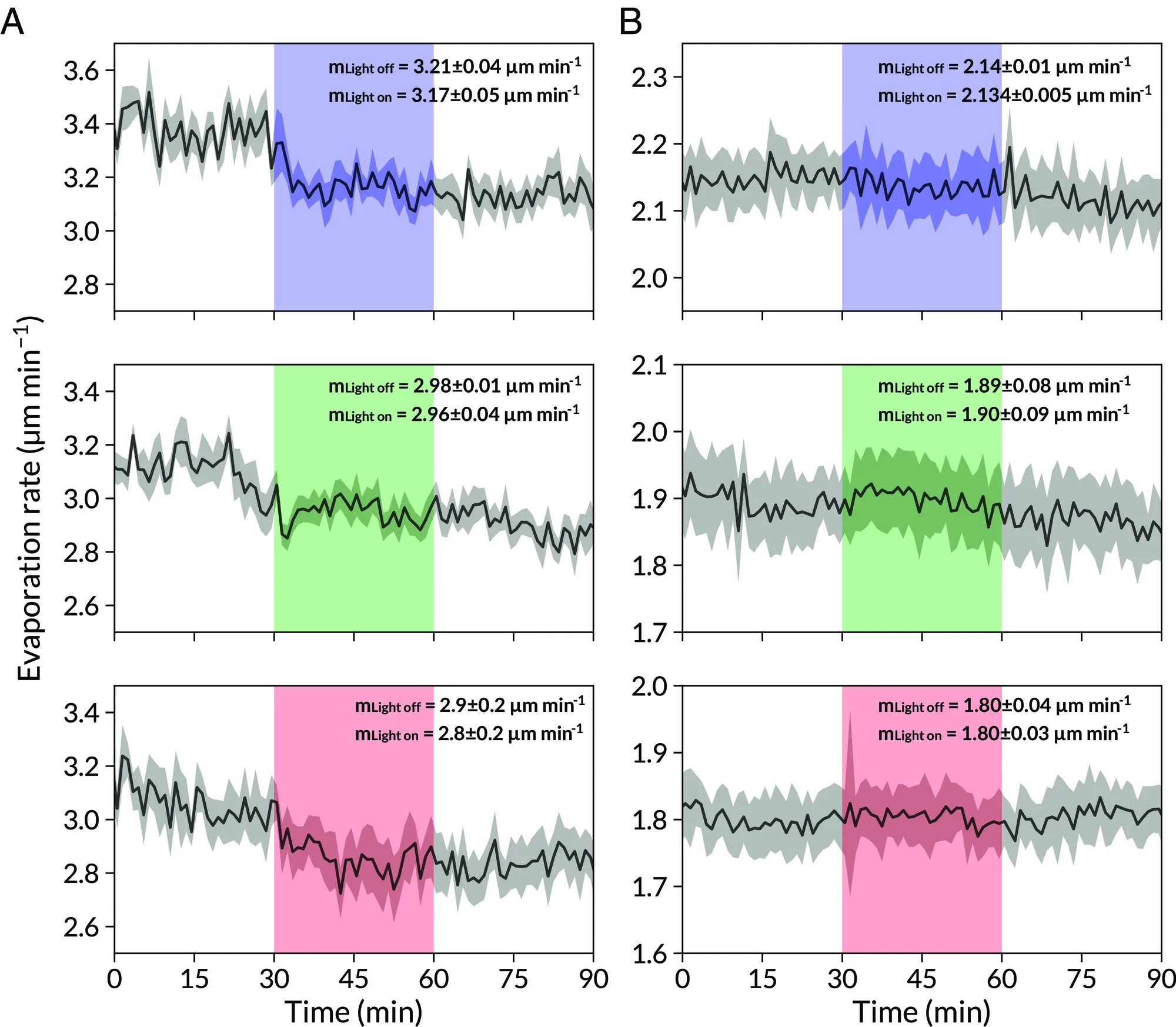
Evaporation rate measured by a confocal displacement sensor under diode laser illumination at (*A*) dry air purged <0.1% RH and (*B*) ~27%, ~33%, ~36% RH for blue, green, and red diode experiments, respectively. The illuminated period is highlighted in the colored region. For the light-off measurements, the reported evaporation rate is an average of the evaporation rates before and after irradiation. The shaded region in each plot represents the period during which the sample is irradiated by the respective light.

To probe whether visible light enhances evaporation and to determine whether this response depends on the illumination wavelength, we irradiated pure Milli-Q water with three continuous-wave diode lasers at 450 nm, 532 nm, and 635 nm. This wavelength range spans the visible region previously reported to exhibit anomalous evaporation behavior, including a pronounced maximum near 520 nm (12, 13). For all measurements, the incident beam was p-polarized [transverse magnetic (TM) polarization] and directed onto the water surface at an angle of incidence of 45°, consistent with the conditions used in the previous reports. All beams were focused to achieve comparable power densities (~1,000 W m^–2^), chosen to match the illumination conditions employed in earlier studies. In addition, as prior interpretations suggested that ambient humidity may influence the interfacial reorganization of photomolecular water clusters (13), measurements were conducted under two controlled atmospheric conditions: a dry air purged environment [<0.1% relative humidity (RH)], and an unpurged room environment (~27%, ~33%, ~36% RH for blue, green, and red diode experiments, respectively). For each wavelength–humidity combination, the experiment consisted of three consecutive time windows: 30 min of “light off,”30 min of “light on,” and a final 30 min of “light off,” thereby allowing us to identify any reversible changes in evaporation rate associated with the presence of visible light. The results are presented in Fig. 2 *A* and *B*. The purged measurements (<0.1% RH; Fig. 2*A*) show that during the initial dark period the evaporation rate shows little to no change with time, reflecting a stable baseline evaporation rate. When the visible beam is introduced, the slope remains relatively unchanged within experimental noise. The evaporation continues at the same linear rate for the full duration of illumination, with no perturbation within our detection limits at any of the three wavelengths. When the light is switched off, the evaporation rate remains identical to both the preillumination and illumination periods. To quantify these observations, evaporation rates were extracted from linear fits to the height-time traces within each 30 min window (Fig. 2), and the relative change under illumination was computed as $(m_{\text{light}}-m_{\text{dark}})/m_{\text{dark}}$. Under dry purged conditions the evaporation rate change ranges from −1.8 to −0.8%. No systematic wavelength dependence is observed.

We next repeated the measurements under unpurged RH conditions (~27%, ~33%, ~36% for blue, green, and red diode experiments, respectively, Fig. 2*B*), matching the ambient environments used in earlier visible-illumination studies. If humidity were to play a decisive role in mediating any light-induced response (13), one might expect that visible irradiation would produce measurable deviations from the baseline evaporation slope specifically under these ambient conditions. However, the unpurged measurements show the same behavior as observed under dry conditions (Fig. 2*B*). Under unpurged RH conditions, the relative change ranges from −0.1 to 0.7% across wavelengths. Within the humidity range explored here, visible irradiation does not measurably alter the evaporation rate of neat water.

To evaluate whether the absence of a detectable effect could arise from limited experimental sensitivity, we quantified the precision of our evaporation-rate measurements. The absolute baseline evaporation rate measured here (1.8 to 2.2 μm min^−1^) under ambient conditions is consistent with previously reported values (~1.5 and ~1.2 μm min^–1^) (13, 16), supporting the comparability of the experimental conditions and evaporation regime. The statistical uncertainty of the fitted evaporation slopes, normalized by the baseline rate, corresponds to a relative detection limit of approximately 4.8%. Prior studies, however, reported substantial light-induced enhancements in evaporation rates. In particular, the mass-based measurements by Lv et al. yielded an increase of approximately 12% under illumination conditions comparable to those employed here (13), while interferometric measurements by Verma et al. reported changes of up to approximately 40% under higher light intensities (16). While interferometric approaches offer higher nominal precision, the magnitude of the reported effects in both cases far exceeds our experimental uncertainty; a clear change in evaporation rate between light-off and light-on intervals would manifest as an enhancement of that magnitude, which is not observed.

Across all six measurement conditions, three wavelengths, and two humidity environments, we observe no significant light-induced change in the evaporation rate of neat water. In particular, the previously reported enhancement in evaporation rate at green wavelengths does not appear under our analogous experimental conditions. These findings place the earlier reports in a broader experimental context. Previous studies observed enhanced evaporation or related signatures under visible illumination in hydrogels, porous matrices, or droplet geometries, and interpreted these observations as evidence for a nonthermal, photon-driven perturbation of interfacial water. In those geometries, evaporation must be inferred indirectly and can couple to geometry-dependent heat and mass transport, whereas the present measurements directly track the recession of a flat air–water interface.

Taken together, these measurements show that visible illumination at intensities comparable to solar flux does not measurably enhance evaporation at a flat air–water interface. Any substantial evaporation anomalies reported in structured or porous materials at similar fluxes are more plausibly attributed to properties of those materials or their geometries, rather than from an intrinsic visible-light sensitivity of a flat air–water interface. If a nonthermal mechanism were operative at the interface, it would be expected to produce detectable changes in interfacial structure. To directly test this possibility, we next probe the interfacial hydrogen-bond structure using heterodyne-detected sum-frequency generation (HD-SFG) spectroscopy, which provides direct, surface-specific sensitivity to the orientation and bonding environment of interfacial water molecules.

### HD-SFG Reveals No Modification of Interfacial Water Structure under Visible Irradiation.

While the confocal displacement measurements provide a direct macroscopic assessment of whether visible photons influence the evaporation rate of neat water, they do not, by themselves, rule out the possibility that more subtle structural changes occur at the molecular level. The photomolecular effect, as originally proposed, asserts that visible light perturbs the interfacial hydrogen-bond network, weakens or distorts intermolecular interactions, and in some cases ejects multimolecule clusters directly into the vapor phase. According to this mechanism, even if the resulting evaporation enhancement were small or transient, such perturbations should alter the interfacial water structure. Therefore, to complement the macroscopic evaporation measurements, we performed heterodyne-detected vibrational sum frequency generation (HD-SFG) spectroscopy under identical illumination conditions.

HD-SFG is uniquely suited to probe the molecular structure of the air–water interface. As SFG is forbidden in centrosymmetric media, the signal arises exclusively from the interfacial region where inversion symmetry is inherently broken (34). For the air–water interface, the signal originates from the first few Ångströms, or roughly the first 2 to 3 water layers. The technique is sensitive to the orientation, hydrogen-bonding strength, and local field environment of interfacial OH groups, enabling even subtle changes in intermolecular interactions to be detected (35–37). Importantly, SFG directly probes the very structural motifs that the photomolecular effect claims are modified by visible irradiation: hydrogen-bond stretching, free-OH populations, and orientational ordering (11, 35, 38). If visible light modifies the evaporation rate by perturbing the interfacial hydrogen-bond network, corresponding changes should be observed in the SFG spectrum. A change in hydrogen-bond strength would produce shifts in the O–H stretch vibrational frequencies, whereas changes in the average orientation of interfacial water molecules would affect the SFG amplitude. Furthermore, interfacial restructuring would be expected to alter the intensity of the free-OH peak near 3,700 cm^–1^ and modify the overall spectral line shape (Fig. 1).

**Fig. 3.**
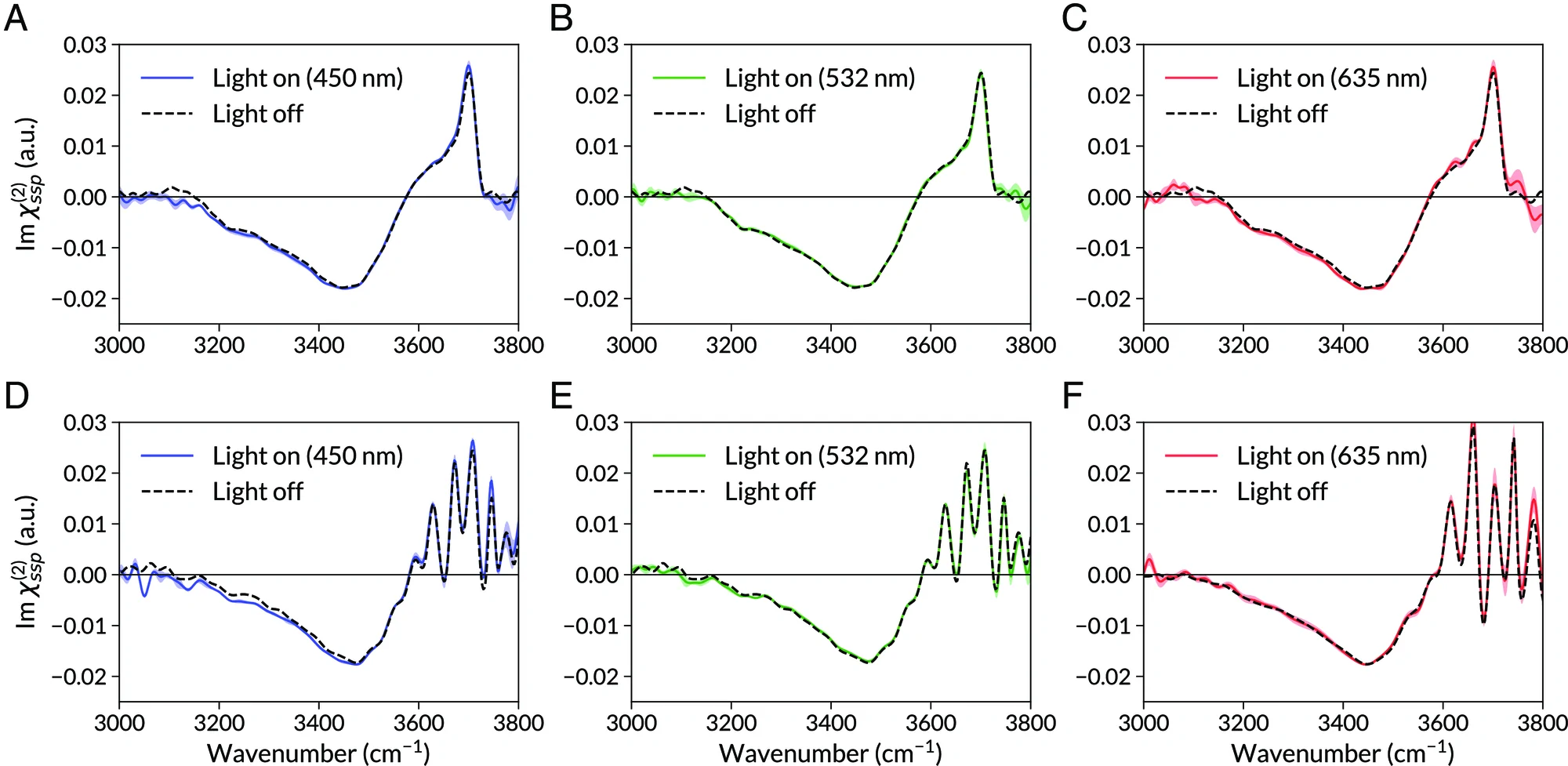
SFG spectra at the ssp polarization combination of the air–water interface under diode laser illumination at a <0.1% humidity condition (*A*–*C*) and a ~45% (*D* and *E*) or ~55% (*F*) room humidity condition (*D*–*F*). The high-frequency (3,600 to 3,800 cm^−1^) oscillations observed under humid conditions are caused by the infrared beam interacting with ambient water vapor, and cannot be assigned to distinct interfacial water structures. Fresnel factor corrected spectra using the Lorentz and Slab models are presented in *SI Appendix*, Fig. S2.

To test this, we recorded HD-SFG spectra of the neat air–water interface in three illumination conditions corresponding to the diode wavelengths used in the evaporation measurements (450 nm, 532 nm, 635 nm) and under both humidity environments (<0.1% RH and ~45%, ~45%, and ~55% RH for blue, green, and red, respectively). Prior to each experiment, the water sample was allowed to equilibrate to the respective humidity conditions before the experiments were conducted (~60 min). In addition to ensuring beam overlap of the diode with the up conversion and MIR beam, all other optical parameters, incident angles, and pulse energies were held constant, ensuring that any observed differences could be attributed solely to the visible irradiation. The results are shown in Fig. 3 *A*–*F*. Across all wavelengths and both humidity conditions, the HD-SFG spectra reveal no measurable change in the OH-stretch region upon illumination. Within the experimental noise level, the bonded OH continuum (3,000 to 3,600 cm^–1^) maintains identical amplitude and curvature under light-on and light-off conditions. The position and shape of the free-OH peak near 3,700 cm^–1^, one of the most sensitive indicators of interfacial perturbation (36, 39, 40), remain unchanged. No systematic shifts, broadening, or intensity variations are observed across the six measurement conditions. The spectra overlay almost perfectly across wavelengths, demonstrating that visible irradiation does not alter the interfacial vibrational response, a highly sensitive reporter of interfacial structure. As each spectrum is compared with its own light-off reference under identical geometry, wavelength, and polarization, any Fresnel or interfacial-dielectric correction is common to the two and cancels in the comparison; the invariance is therefore evident in the raw response and does not rely on any specific dielectric profile or three-layer correction scheme. See *SI Appendix*, *Note S1* for a detailed explanation of Fresnel factor corrections (25, 31, 41–47). See *SI Appendix*, Figs. S2 and S3 for a spectral comparison of each model. Moreover, the photomolecular enhancement is, in its original formulation described previously (32), an intensive interfacial process defined per unit illuminated area; the SFG measurement, whose focus lies within the irradiated region, therefore probes it locally and without the spatial averaging inherent to sample-integrating measurements.

This lack of observable structural modification places important constraints on nonthermal visible-light mechanisms at the air–water interface. As SFG is sensitive to intermolecular interactions and orientational order, the spectral regions shown in Fig. 1*B* provide direct markers of interfacial structure: the broad hydrogen-bonded OH continuum (≈3,000 to 3,600 cm^–1^) reflects the strength and connectivity of the interfacial hydrogen-bond network, while the narrow free-OH feature (≈3,700 cm^–1^) reports on undercoordinated OH groups oriented toward the vapor phase. Perturbations that weaken or disrupt the hydrogen-bond network—such as increased temperature, curvature, or ion adsorption—typically produce a blue shift and reduced amplitude of the hydrogen-bonded continuum, along with changes in the free-OH intensity due to altered orientational populations. As a concrete benchmark, molecular dynamics simulations across interfaces of differing curvature show that changing the interfacial dangling-OH fraction from 6 to 18% between cavity, slab, and droplet geometries, produces a pronounced, quantifiable change in the free-OH vibrational density of states. This confirms that a defined change in interfacial hydrogen-bond structure maps onto a defined spectroscopic change (15). Consequently, perturbations large enough to eject multimolecule clusters would be expected to involve partial hydrogen-bond breaking, reorientation of OH groups, or stabilization of non-bulk-like configurations, all of which should manifest as measurable changes in spectral amplitude, frequency, or lineshape (10, 35, 48). Across all illumination conditions examined here, including excitation at 532 nm, where prior studies reported the strongest response, no such modifications are detected. The invariance of the HD-SFG spectra across humidity conditions further indicates that relative humidity does not induce or activate any detectable interfacial response under visible illumination. Within the wavelength, intensity, and humidity ranges explored here, the interfacial hydrogen-bond network of neat interfacial water remains unchanged.

When considered alongside the macroscopic evaporation measurements, the HD-SFG results establish a consistent picture across length scales. The absence of any molecular-level perturbation under visible irradiation is fully consistent with the observed invariance of the evaporation rate, as changes in evaporation flux would typically be expected to involve modification of interfacial structure. Moreover, evaporation is itself a molecular, interfacial process, in which a water molecule departs by breaking its hydrogen bonds to the outermost layer, thus the rate is governed by the same interfacial hydrogen-bond connectivity and free-OH population that HD-SFG reports directly (9). This structural control of evaporation is established experimentally: interfacial hydrogen-bond ordering measured by surface-specific spectroscopy tracks the evaporation rate across Hofmeister salts and the nonideal vapor pressures of water–alcohol mixtures (10, 11). As such, a spectrally invariant interface is still informative about evaporation; it serves as independent evidence that the interfacial structure governing the rate is unperturbed. The interface is highly dynamic and self-heals on ultrafast timescales, therefore a rare, rapidly healed perturbation would not register in a time-averaged measurement; the mechanism under test, however, must sustain cluster ejection under continuous illumination, and therefore requires a correspondingly sustained steady-state population of perturbed interfacial OH groups rather than a transient one. This steady-state ensemble is precisely what the time-averaged spectrum integrates, therefore for a continuously driven process the invariance we observe is the matched observable and constrains any sustained interfacial perturbation. The combined structural and kinetic measurements thus provide a quantitative benchmark for the response of the neat air–water interface to visible light. Within the experimentally accessible regime investigated here, no evidence is found for a nonthermal visible light–driven modification of interfacial water or evaporation dynamics.

### High-Peak-Power Illumination Leaves Interfacial Water Unchanged.

The continuous-wave diode illumination experiments above probe the response of the neat air–water interface under modest average power densities, but a null result under these conditions does not by itself exclude all possible nonthermal effects. In particular, if the proposed photomolecular mechanism arises from a field-induced perturbation of the interfacial hydrogen-bond network rather than simple heating, its signature should scale primarily with instantaneous electric-field strength rather than average absorbed power. To test this directly, we performed additional HD-SFG measurements using high-peak-power upconversion pulses at 515 nm, 800 nm, and 1030 nm, delivering instantaneous photon fluxes many orders of magnitude higher than those in the continuous-wave experiments. This provides a stringent test of a nonthermal, field-driven mechanism: if visible light weakens intermolecular interactions or promotes cluster ejection through direct field coupling, the resulting perturbation should become more apparent in the interfacial vibrational response under these conditions. We note, however, that nearly symmetric or ultrafast-relaxing transient structures, subdegree temperature increases, or modest uncertainties in Fresnel and dielectric corrections could reduce the observable spectral response. Even with these caveats, the absence of detectable changes under extreme peak intensities places strong constraints on any nonthermal, field-induced restructuring of the neat air–water interface.

The 515 nm, 800 nm, and 1030 nm upconversion pulses used here span a broad range of pulse energies and repetition rates. For the 515 nm beam (0.9 μJ at 50 kHz; ~45 mW average power), 800 nm beam (8 μJ at 1 kHz; ~8 mW average power), and 1030 nm beam (1.4 μJ at 50 kHz; ~70 mW average power), the corresponding pulse durations were approximately >2.6 ps, ~1 ps, and ~2.6 ps, respectively. These parameters yield estimated peak power densities of 1.6 × 10^10^ W cm^−2^ (515 nm), 2.3 × 10^10^ W cm^−2^ (800 nm), and 2.1 × 10^10^ W cm^−2^ (1030 nm). For comparison, the green diode illumination used earlier has a peak power density of ~0.1 W cm^−2^, over 10 orders of magnitude lower than that of the pulsed excitation employed here. Thus, these experiments probe the photomolecular hypothesis under conditions that strongly amplify any putative photon-flux-dependent response.

**Fig. 4.**
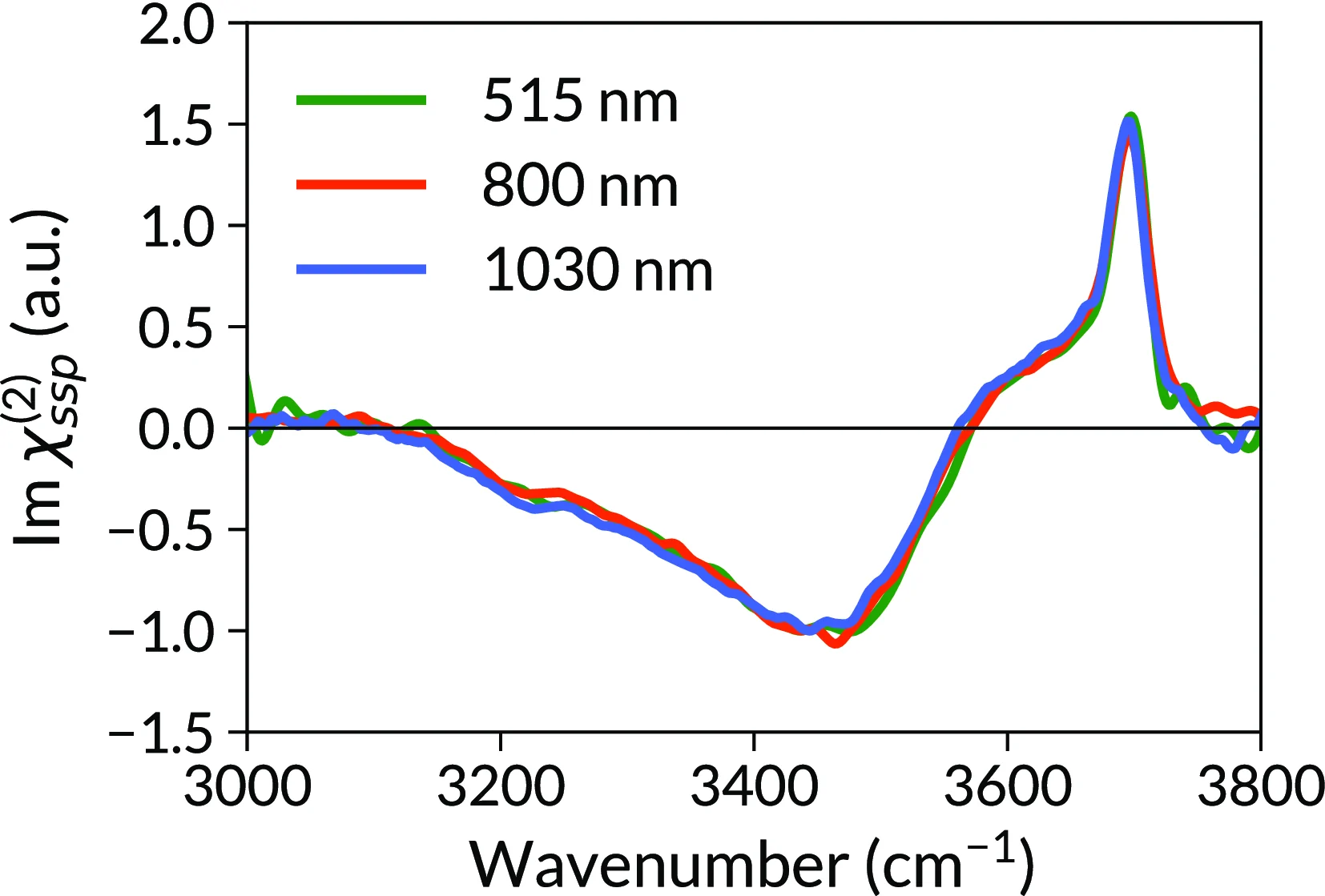
The Im χ^(2)^ spectra at the ssp polarization combination of the neat air–water interface recorded with 515 nm (green), 800 nm (red), and 1030 nm (blue) upconversion pulses. Spectra were normalized by their respective H-bonded intensity. Fresnel factor corrected spectra using the Lorentz and Slab models are presented in *SI Appendix*, Fig. S3. Despite peak power densities exceeding 10^10^ W cm^–2^, the spectral amplitude, lineshape, and free-OH response remain indistinguishable for the three upconversion wavelengths. The absence of any detectable spectral change establishes that even extreme instantaneous photon flux does not perturb the interfacial hydrogen-bond network.

Fig. 4 shows the resulting HD-SFG spectra obtained under illumination with each of the three upconversion pulses. To ensure a direct comparison across excitation wavelengths, each spectrum was normalized to its respective hydrogen-bonded OH band. To confirm that this cross-wavelength comparison does not depend on a particular dielectric model, the same spectra were also processed using the wavelength-dependent Fresnel correction with two profiles that treat the interfacial dielectric constant differently, the Lorentz and slab models; all three approaches, Lorentz-corrected, slab-corrected, and bonded-OH-normalized, are spectrally invariant (*SI Appendix*, Fig. S3). Across the entire OH-stretch region, including the hydrogen-bonded continuum (3,000 to 3,600 cm^–1^) and the free-OH mode (~3,700 cm^–1^), no measurable changes in spectral amplitude, peak position, or lineshape are observed. The spectra recorded under pulsed illumination are indistinguishable, within experimental sensitivity, from those acquired in the absence of illumination. No systematic broadening, shift, or intensity modulation is detected at any of the three wavelengths. The absence of any detectable structural response at peak power densities exceeding 10^10^ W/cm^2^ places strong constraints on proposed nonthermal visible-light mechanisms acting at the neat air–water interface. Within the wavelength, intensity, and timescale ranges explored here, the interfacial second-order response remains invariant, indicating that visible irradiation does not measurably perturb interfacial hydrogen bonding even under extreme instantaneous photon flux. As the three upconversion pulses derive from different laser systems (for example, 2 µJ of MIR at 1 kHz for the 800 nm spectrometer vs. 0.4 µJ of MIR at 50 kHz for the 515 nm/1030 nm spectrometer), they differ substantially in the pulse energy, repetition rate, and peak power of both the MIR and upconversion pulses. The spectral invariance therefore additionally rules out any structural perturbation by the probe pulses themselves, including the resonant mid-infrared pulse. Moreover, the approximately 100 to 200 fs IR probe pulses are separated by microseconds to milliseconds, allowing any pulse-induced perturbation to relax before the next pulse arrives. Thus, SFG samples a near-instantaneous interfacial configuration rather than driving rearrangement on the timescale of continuous-wave illumination.

### Contextualizing Prior Observations of Visible Light–Driven Evaporation.

The present results clarify the conditions under which visible illumination can influence observables associated with water evaporation and interfacial response. In systems such as porous hydrogels, polymer matrices, open-air optical geometries, or mechanically sensitive droplet configurations, measured signals can be affected by factors including optical absorption and scattering, internal heat transport, local vapor concentration, and radiation-pressure forces. These contributions can modulate indirect observables, such as mass loss, above-surface spectra, or droplet shape, without requiring a corresponding change in the molecular structure or kinetics of a planar air–water interface.

By contrast, the combination of direct interface-height tracking and heterodyne-detected SFG spectroscopy employed here isolates both macroscopic evaporation flux and molecular-level structure at a well-defined, planar air–water interface. Across ambient and dry conditions, visible wavelengths spanning those used in previous work, and instantaneous photon fluxes exceeding earlier experiments by several orders of magnitude, neither evaporation kinetics nor the interfacial response exhibits any measurable dependence on illumination. These results place quantitative constraints on the proposed nonthermal visible-light mechanisms at water surfaces and suggest that any substantial light-induced evaporation anomalies reported in structured or porous media are more consistent with photothermal or geometry-dependent effects in complex materials than with a nonthermal pathway intrinsic to liquid water.

## Conclusions

The experiments presented here combine direct macroscopic evaporation measurements, surface-specific vibrational spectroscopy, and extreme photon-flux tests to evaluate the response of the neat air–water interface to visible illumination. Across all approaches, we find that the evaporation rate of a flat-water surface remains linear and unchanged under continuous illumination at 450 nm, 532 nm, and 635 nm, independent of ambient humidity. Complementary surface-sensitive spectroscopy shows that the interfacial OH-stretch spectrum is invariant under identical conditions, with no changes in amplitude, frequency, or lineshape of either the hydrogen-bonded continuum or the free-OH mode. This spectral invariance indicates that visible light does not measurably perturb the orientational order or interfacial hydrogen-bond network of interfacial water under these conditions.

To assess whether such effects arise only at extremely high instantaneous intensities, we exposed the interface to ultrafast upconversion pulses across the visible and near-infrared range, delivering peak power densities many orders of magnitude greater than those of continuous-wave excitation. Even under these extreme excitation conditions, the interfacial vibrational response remains unchanged. Within the ranges of wavelength, intensity, humidity, and timescale explored here, visible irradiation produces no modification of interfacial structure or evaporation kinetics, establishing quantitative upper bounds on any nonthermal visible-light mechanisms at neat water surfaces within our experimental sensitivity.

Beyond resolving a specific controversy, these findings provide a robust benchmark for light–water interactions at interfaces and supply clear physical constraints for the design and interpretation of light-driven evaporation and water-harvesting technologies. They show that substantial visible-light-induced evaporation enhancements must arise from photothermal or geometric effects in complex, absorbing materials rather than from a new photophysical pathway intrinsic to liquid water. More broadly, the demonstrated insensitivity of the air–water interface to visible photons highlights the remarkable stability of interfacial hydrogen bonding and offers a well-defined reference system against which interfacial responses in more complex chemical or environmental scenarios can be quantified.

## Methods

Evaporation rates were determined using height displacements from a Keyence CL-L070 Series Confocal Displacement Sensor (7 cm working distance), with submicron relative precision (<0.25 μm resolution) and manufacturer-specified linearity of ±2 μm over the measurement range. Measurements were carried out under two humidity conditions: i) dry air purged, resulting in a relative humidity < 0.1%, and ii) ambient relative humidity conditions (~27%, ~33%, ~36% RH for blue, green, and red diode experiments, respectively). The temperature was kept constant throughout each measurement (~22.1 °C). Three diode lasers with center wavelengths of 450 nm (Thorlabs CPS450), 532 nm (Thorlabs CPS532), and 635 nm (Thorlabs CPS635), and an output power of 4.5 mW, were used as light sources. The output of each laser was focused onto the sample with a 10 cm plano-convex calcium fluoride lens, achieving a power density of ~1,000 W/m^2^ at the water surface. This corresponds to an irradiated area of 4.5 mm^2^ (~2.4 mm diameter) at the water surface, within which the confocal sensor samples a 0.6 mm diameter region (0.28 mm^2^) located concentrically inside the illuminated spot. The incident angle of each diode laser was fixed at 45°, and the incident beam was p-polarized (TM) using a Glan laser calcite polarizer. A diagram of the setup can be found in *SI Appendix*, Fig. S1. A 2 cm diameter glass trough containing Milli-Q water (18.2 MΩ cm resistivity) was mounted on a translational stage (Thorlabs MLJ250). Prior to data collection, the sample was equilibrated at room temperature under the selected humidity condition for 30 min to ensure steady-state evaporation. Height data were then recorded in three sequential intervals: 30 min with the lasers off, 30 min with the lasers on, and 30 min with the diodes off again. Height data were collected at 100 ms intervals; for each time point, the evaporation rate was computed as the slope of a linear fit to the nearest 600 points to suppress short-timescale noise while preserving long-term trends.

The samples were subsequently measured using a heterodyne-detected vibrational sum-frequency generation spectrometer. All spectrometer parameters relevant to the present measurements are summarized here to ensure reproducibility (31). Briefly, a 1030 nm Yb:KGW diode-pumped solid-state amplifier laser source (Pharos, Light Conversion), with a pulse energy of 200 µJ, operating at 50 kHz, was used as the primary light source. The subsequent output radiation was split into two portions: one portion was spectrally narrowed to ~7 cm^–1^ and used as the upconversion beam, while the other portion was directed into a bespoke OPA-DFG system to generate broadband mid-IR light with a bandwidth (1/e^2^) of ~800 cm^–1^, spanning 3,000 to 4,000 cm^–1^. The spectrally narrowed visible and broadband mid-IR beams were then combined collinearly and focused onto a quartz plate to generate a local oscillator (LO) signal. A 5 mm thick SrTiO_3_ window was inserted into the beam path before the sample to impose a ~2 ps delay between the signal and LO response. The LO signal, together with the visible and mid-IR pulses, was subsequently focused onto the sample at an incident angle of 53°. The resulting SFG signal interfered with the LO, producing an interferogram that was directed into a spectrometer (Andor Kymera 328i) and detected by a thermoelectrically cooled CCD camera (Andor iDus 420) operated at −100 °C. To ensure spatial overlap of the visible, mid-IR, and LO beams with the diode lasers, a pinhole was employed. The 80 µm diameter SFG probe (0.005 mm^2^) lies entirely within the diode-irradiated spot; thus, the spectra report exclusively on illuminated interface. For measurements employing a 515 nm upconversion pulse, the narrowband 1030 nm beam was frequency-doubled in a BBO crystal. The optical layout remained identical to that described above, except that a 0.5 mm thick SrTiO_3_ window was used to generate the 2 ps LO delay.

A different spectrometer was utilized for experiments employing an 800 nm upconversion pulse. The specifications of this additional spectrometer have been detailed in previous studies (36, 37, 48). In this configuration, SFG measurements were carried out in a collinear beam geometry using a Ti:Sapphire laser system (Spectra-Physics; ~40 fs pulse duration, 5 mJ pulse energy, 1 kHz repetition rate). A portion of the output was directed to a pulse shaper to generate a narrowband visible pulse (centered at ~800 nm, bandwidth ~15 cm^–1^), while the remaining output pumped a commercial optical parametric amplifier (TOPAS SHBS-400, Spectra-Physics) to produce a broadband mid-IR pulse (centered at ~3,000 cm^–1^, bandwidth ~350 cm^–1^). The visible and mid-IR pulses were first focused onto a y-cut quartz crystal to generate a local oscillator and subsequently onto the water surface at an incident angle of 45° after passing through a 2 mm thick SrTiO_3_ plate. The resulting SFG signal from the water surface interfered with the LO to generate an interferogram, which was recorded using a liquid-nitrogen-cooled CCD camera (Teledyne Princeton Instruments, PyLoN).

## Supplementary Material

Appendix 01 (PDF)

## Data Availability

Data supporting the findings of this study have been deposited in Edmond Open Research Data Repository (DOI: 10.17617/3.Q2GHPJ) (49).
