# Supplementary material for: Visible light leaves evaporation and interfacial structure of neat water unchanged at the air–water interface
Source: Proc Natl Acad Sci U S A. 2026 Sep 14;123(38):e2615377123. doi: 10.1073/pnas.2615377123 (PMC13598155; doi:10.1073/pnas.2615377123)
Supplement: Supplementary file 1 [file pnas.2615377123.sapp.pdf]

PNAS

Supporting Information for

Visible light leaves evaporation and interfacial structure of neat water unchanged at the air–water interface.

Yucong Chen, Joseph C. Shirley, Zi Xuan Ng, Yongkang Wang, Yuki Nagata, Arsh S. Hazrah*, Mischa Bonn*

Arsh S. Hazrah, Mischa Bonn.
Email: hazraha@mpip-mainz.mpg.de, bonn@mpip-mainz.mpg.de

This PDF file includes:

Note S1. Fresnel Factor Corrections
Figure S1. Confocal Displacement Sensor
Figure S2. Fresnel Factor Corrected SFG spectra of air–water interface under diode laser illumination
Figure S3. Cross-wavelength comparison of Im $\chi^{(2)}$ spectra under Fresnel and normalization treatments
References

Note S1. Fresnel Factor Corrections

Fresnel factor corrections are applied to enable direct quantitative comparison of SFG spectra obtained with different up-conversion wavelengths. This procedure removes wavelength-dependent optical effects, ensuring that observed spectral differences reflect an intrinsic interfacial response. The formalism has been established previously and is only briefly summarized here.[1–3] Wavelength-dependent complex refractive indices for air, quartz, and water were obtained for all fundamental, mid infrared, and SFG wavelengths. For air, the complex refractive index was set to 1+0i and not a function of wavelength. Refractive indices for water and quartz were collected from refractiveindex.info,[4] and ultimately from Hale[5] and Franta,[6] respectively. Given the refractive indices of medium I (air) and medium II (water or quartz) the interfacial refractive index was calculated using a Lorentz model ($\phi = \frac{\pi}{2}$).[2,7] In Figure S3 we provide a comparison between the Slab and Lorentz model, which show no change between different upconversion wavelengths. The frequency dependent interfacial refractive index, $n(\omega)_{\text{int}}$, is given by:

$$n(\omega)_{\text{int}} = \sqrt{\frac{1 + \left(n(\omega)_{mII}^2 - 1\right) * \frac{2\pi - \phi}{6\pi}}{1 + \frac{1 - n(\omega)_{mII}^2}{6n(\omega)_{mII}^2} * \left(1 + \cos\left(\frac{\phi}{2}\right)\right)^2 * \left(2 - \cos\left(\frac{\phi}{2}\right)\right)}} \qquad \text{Eq. S1}$$

where $n(\omega)_{mII}$ the frequency dependent refractive index of medium II and ϕ is the geometric angle between the surface normal and the line connecting the center of the chromophore sphere to the point of intersection with the dielectric boundary, as defined in Yu et al.[7]

The physical geometry of the SFG instruments described herein are all in a collinear geometry, where the 515 nm, 800 nm, and 1030 nm up conversion pulses propagate at an incident angle of ~53°, ~45°, and ~53°, respectively, with respect to the normal of the sample plane.
Once the interfacial refractive indices and transmission angles are calculated, the Fresnel coefficients $(L_{xx}(\omega), L_{yy}(\omega), L_{zz}(\omega))$ can be determined.

$$L_{xx}(\omega) = \frac{2n(\omega)_{mI} * \cos(\theta(\omega)_{tran})}{n(\omega)_{mI} * \cos(\theta(\omega)_{tran}) + n(\omega)_{mII} * \cos(\theta(\omega)_{inc})} \qquad \text{Eq. S6}$$

$$L_{yy}(\omega) = \frac{2n(\omega)_{mI} * \cos(\theta(\omega)_{inc})}{n(\omega)_{mI} * \cos(\theta(\omega)_{inc}) + n(\omega)_{mII} * \cos(\theta(\omega)_{tran})} \qquad \text{Eq. S7}$$

$$L_{zz}(\omega) = \frac{2n(\omega)_{mII} * \cos(\theta(\omega)_{inc}) * \left(\frac{n(\omega)_{mI}}{n(\omega)_{int}}\right)^2}{n(\omega)_{mI} * \cos(\theta(\omega)_{tran}) + n(\omega)_{mII} * \cos(\theta(\omega)_{inc})} \qquad \text{Eq. S8}$$

Here $\theta(\omega)_{\text{tran}}$ is the frequency dependent angle of the transmitted beam, θ_{inc} is the frequency independent angle of incident beam. The Fresnel coefficients are calculated for both the air – water sample interface and the air–quartz reference interface. In our heterodyne experiments, we use an LO-before-sample geometry, which introduces a −π/2 phase shift that must be applied between the bulk reference and the interfacial sample during the referencing process.[8] To scale the spectra, a quantitative value of the $\chi_{ref}^{(2)}$ response for quartz is used. We use the value 6×10^{-13} m/V based on recency.[1,9] With these parameters defined, the experimentally measured ssp laboratory-frame response, where the polarization labels refer to the SFG, up-conversion, and MIR beams, respectively, can be transformed into the yyz frame, yielding a phase-resolved, quantitatively scaled interfacial $\chi^{(2)}$. In this convention, s-polarized light is perpendicular to the plane of incidence and p-polarized light lies within it. In the molecular reference frame, z denotes the surface normal while x and y span the interfacial plane. Consequently, the p polarization projects onto both x and z components, whereas the s polarization projects exclusively onto y.

$$\chi_{yyz,int,sample}^{(2)} = \frac{\chi_{ssp,int,measured,sample}^{(2)}}{\sigma * \sin\left(\theta_{inc,MIR}\right) * L_{yy,SFG}(\omega) * L_{yy,up} * L_{zz}(\omega)_{MIR}} \qquad \text{Eq. S9}$$

$$\chi^{(2)}_{yyz,bulk,ref} = \frac{\chi^{(2)}_{ssp,measured,ref}}{\sigma * \cos(\theta_{inc,MIR}) * L_{yy,SFG}(\omega) * L_{yy,up} * L_{xx}(\omega)_{MIR} * l_c(\omega)} \quad \text{Eq. S10}$$

$$\chi^{(2)}_{yyz,sample} = \frac{\chi^{(2)}_{yyz,int,sample}}{\chi^{(2)}_{yyz,bulk,ref}} * \chi^{(2)}_{ref} * \left(\cos\left(-\frac{\pi}{2}\right) + i\sin\left(-\frac{\pi}{2}\right)\right) \quad \text{Eq. S11}$$

Here σ is the multiplicity ($\sigma = 1$ for water and $\sigma = 2$ for quartz) and $l_c(\omega)$ is the frequency dependent coherence length. $\chi^{(2)}_{yyz,int,sample}$ is the second order nonlinear response arising from the interface of the sample in the molecular frame (unitless). $\chi^{(2)}_{ssp,int,measured,sample}$ and $\chi^{(2)}_{ssp,measured,ref}$ are the experimentally measured effective nonlinear responses under ssp polarization for the sample and the reference, respectively. $\chi^{(2)}_{yyz,bulk,ref}$ is the second order nonlinear response arising from the bulk of the reference in the molecular frame (unitless). Finally, $\chi^{(2)}_{yyz,sample}$ is the second order nonlinear response of the sample in units of m^2V^{-1}.

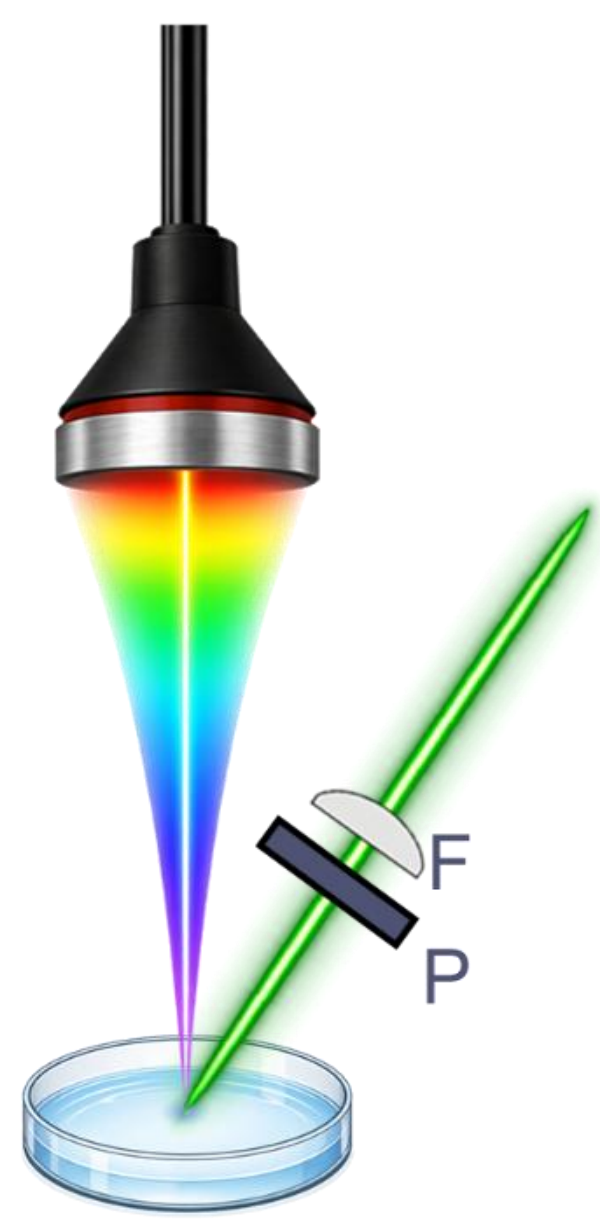

Figure S1. A diagram of the confocal displacement sensor measurements. Planoconvex lens with a focal length of 10 cm (F) and a polarizer (P).

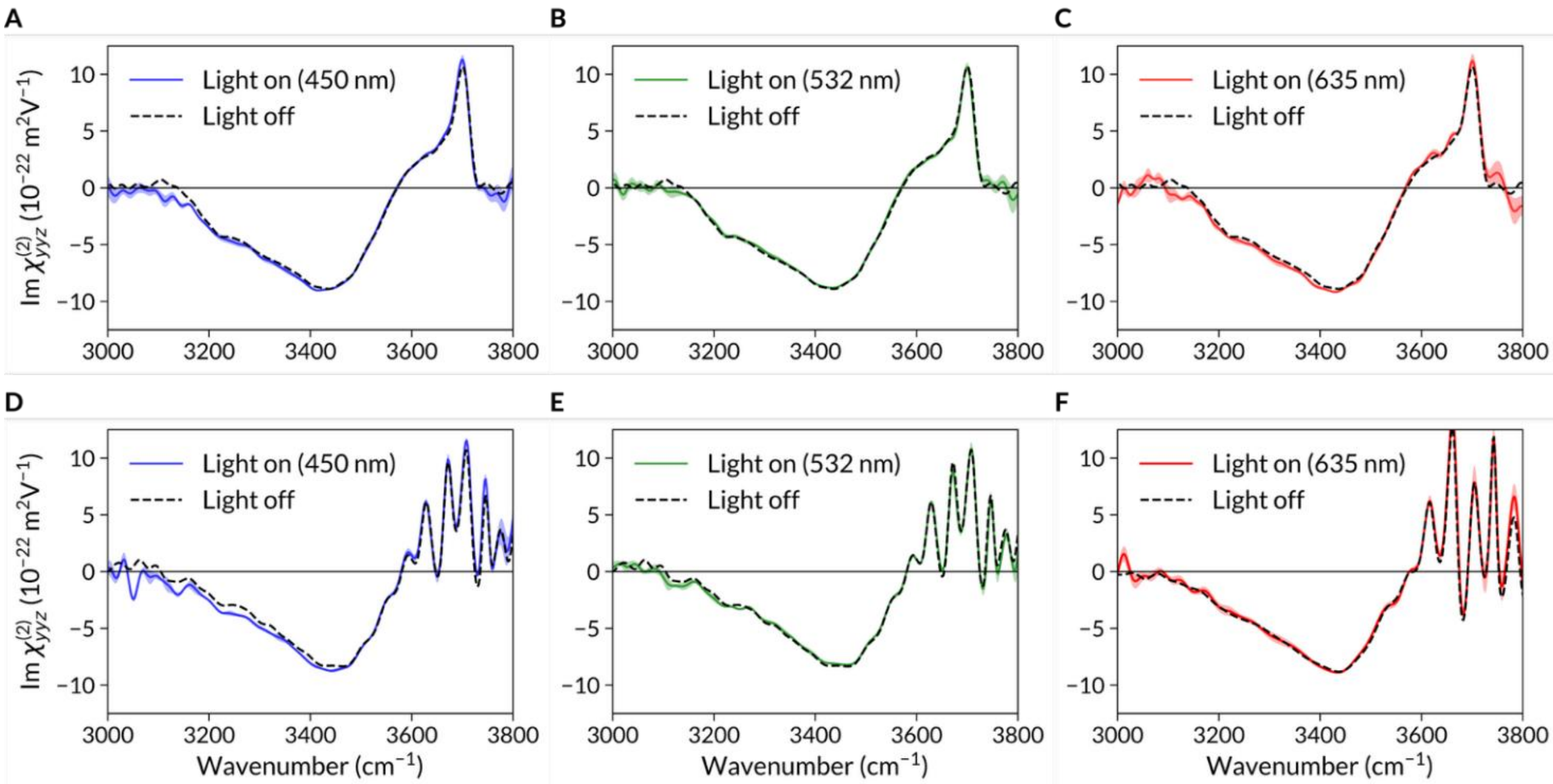

Figure S2. SFG spectra of air–water interface under diode laser illumination at a <0.1% humidity condition (A-C) and a ~45% (D, E) or ~55% (F) room humidity condition (D-F). The high-frequency (3600-3800 cm^{-1}) oscillations observed under humid conditions are caused by the infrared beam interacting with ambient water vapor, and cannot be assigned to distinct interfacial water structures. Spectra were corrected using the Lorentz model.

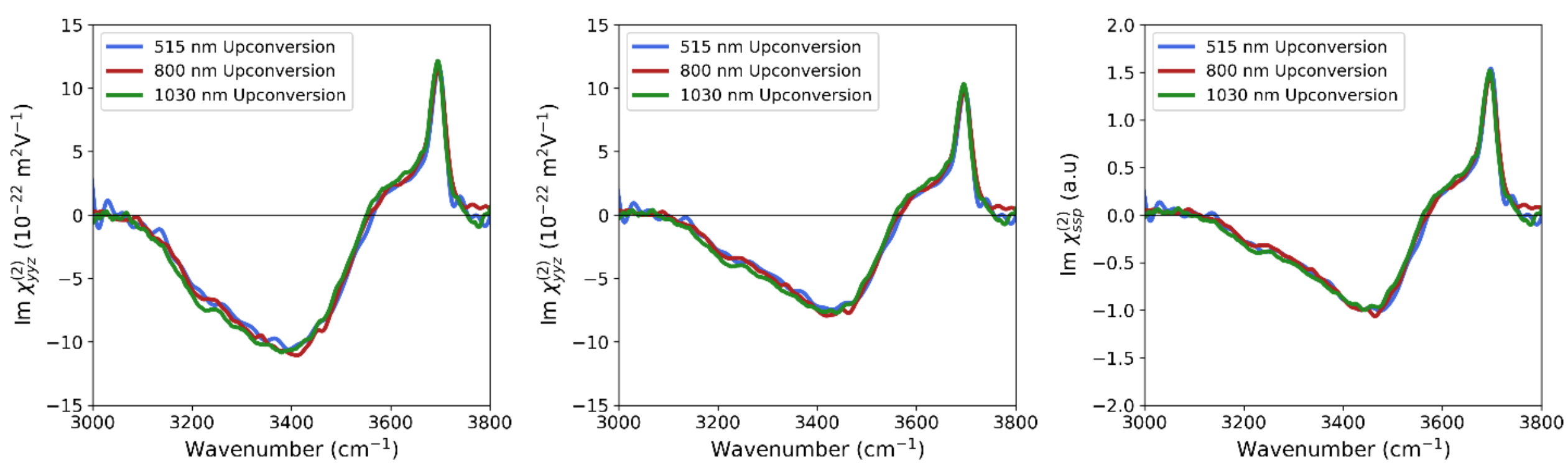

Figure S3. Cross-wavelength comparison of Im $\chi^{(2)}$ spectra under Fresnel and normalization treatments. Left: Fresnel-corrected spectra using the Lorentz dielectric profile. Middle: Fresnel-corrected spectra using the slab dielectric profile. Right: spectra normalized to the hydrogen-bonded OH band, without Fresnel correction. All three treatments show consistent spectral lineshapes and relative peak intensities across the 515 nm, 800 nm, and 1030 nm upconversion wavelengths, confirming that the cross-wavelength comparison in Figure 4 does not depend on the choice of dielectric model or correction method.

References

1 J. C. Shirley, Z. X. Ng, K.-Y. Chiang, Y. Nagata, Y. Litman, A. S. Hazrah and M. Bonn, *J. Am. Chem. Soc.*, 2025, **147**, 46163–46173.
2 X. Yu, K.-Y. Chiang, C.-C. Yu, M. Bonn and Y. Nagata, *J. Chem. Phys.*, 2023, **158**, 044701.
3 K.-Y. Chiang, T. Seki, C.-C. Yu, T. Ohto, J. Hunger, M. Bonn and Y. Nagata, *Proc. Natl. Acad. Sci.*, 2022, **119**, e2204156119.
4 M. N. Polyanskiy, *Sci. Data*, 2024, **11**, 94.
5 G. M. Hale and M. R. Querry, *Appl. Opt.*, 1973, **12**, 555–563.
6 D. Franta, D. Nečas, I. Ohlídal and A. Giglia, in *Optical Micro- and Nanometrology VI*, SPIE, 2016, vol. 9890, 253–267.
7 C.-C. Yu, T. Seki, Y. Wang, M. Bonn and Y. Nagata, *Phys. Rev. Lett.*, 2022, **128**, 226001.
8 S. Nihonyanagi, J. A. Mondal, S. Yamaguchi and T. Tahara, *Annu. Rev. Phys. Chem.*, 2013, **64**, 579–603.
9 A. P. Fellows, Á. D. Duque, V. Balos, L. Lehmann, R. R. Netz, M. Wolf and M. Thämer, *Langmuir*, 2024, **40**, 18760–18772.
